# When the Cough Does Not Improve: A Review on Protracted Bacterial Bronchitis in Children

**DOI:** 10.3389/fped.2020.00433

**Published:** 2020-08-07

**Authors:** Marcella Gallucci, Melissa Pedretti, Arianna Giannetti, Emanuela di Palmo, Luca Bertelli, Andrea Pession, Giampaolo Ricci

**Affiliations:** Department of Paediatrics, S. Orsola-Malpighi Hospital, University of Bologna, Bologna, Italy

**Keywords:** protracted bacterial bronchitis, children, chronic cough, bronchiectasis, airway inflammation

## Abstract

Chronic cough is defined as a daily cough that persists longer than 4 weeks. Protracted bacterial bronchitis (PBB) is a common cause of chronic wet cough in preschool children with no symptoms or signs of other specific causes, and resolution usually follows a 2-week course of an appropriate oral antibiotic. The diagnosis is mainly clinical; generally, no instrumental examinations are necessary. The most common bacteria found in the bronchoalveolar lavage (BAL) of subjects with PBB include *Haemophilus influenzae, Streptococcus pneumoniae*, and *Moraxella catarrhalis*. Nowadays, there is no certain evidence of the role of viruses in PBB pathogenesis even though different types of viruses have been detected in BAL from children with PBB. Airway malacia is commonly found in children with PBB; conversely, there is no correlation with any type of immunodeficiency. Amoxicillin-clavulanate acid is the most commonly used antibiotic, as first-line, prolonged therapy (longer than 2 weeks) is sometimes required to cough resolution. When the wet cough does not improve despite prolonged antibiotic treatment, an underlying disease should be considered. Moreover, there are several hypotheses of a link between PBB and bronchiectasis, as recent evidences show that recurrent PBB (>3 episodes/years) and the presence of *H. influenzae* infection in the lower airways seem to be significant risk factors to develop bronchiectasis. This underlines the importance of a close follow-up among children with PBB and the need to consider chest computerized tomography (CT) in patients with risk factors for bronchiectasis. In this brief review, we summarize the main clinical and pathogenetic findings of PBB, a disease that may be related to a relevant morbidity and decreased quality of life during the pediatric age.

## Background

According to the European, American, and Australian guidelines, chronic cough is defined as a daily cough that persist longer than 4 weeks (1–4).

Conversely, British authors define a chronic cough as a cough longer than 8 weeks; the main reason to classify cough in this way is that a period of 3-4 weeks enables the resolution of the most common infective causes of cough while allowing to identify those children who might require further investigations (5). Nevertheless, the British Thoracic Society (BTS) guidelines were last updated in 2008, while the suggested duration of chronic cough is currently considered 4 weeks.

Chronic cough in childhood is related to a considerable morbidity and a decreased quality of life (QoL) scores, affecting the child's sleep, the ability to play, and the school performance (6). It may also cause a state of anxiety for parents.

Nevertheless, the real impact of chronic cough on QoL is difficult to quantify (6). Both generic health-related (PedsQL) and chronic cough-specific (PC-QoL) QoL scores among children with PBB are similar to those of children with other respiratory disease such as asthma or bronchiectasis (1).

Etiologies of chronic cough include several and heterogenous disease such as asthma, upper airway cough syndrome, and protracted bacterial bronchitis (PBB) (7).

Chronic wet cough may also be a symptom of a chronic suppurative airway disease including bronchiectasis (8).

PBB clinical condition was first described by Marchant et al. in an Australian study among children with a history of chronic wet cough lasting more than 4 weeks, a positive culture of a respiratory pathogen on BAL (bacterial growth ≥10^4^ CFU/ml in BAL) obtained during a flexible bronchoscopy and a clinical response to 2 weeks treatment with antibiotics (amoxicillin-clavulanate acid) (9) (Table 1). Currently, this definition has been reclassified as PBB-micro, and new diagnostic criteria have been developed on the basis of clinical symptoms, thus eliminating the need for BAL, not performed routinely among the pediatric population.

**Table 1:** Protracted bacterial bronchitis (PBB) diagnostic criteria.

	**Diagnostic criteria**
PBB-Clinical	1) Continuous chronic (>4 weeks' duration) wet or productive cough 2) Absence of symptoms or signs suggestive of other causes of wet or productive cough 3) Cough resolved following a 2-week course of an appropriate oral treatment
PBB-micro	1) History of chronic wet cough lasting more than 4 weeks 2) Positive culture of a respiratory pathogen on BAL (bacterial growth ≥10^4^ CFU/ml in BAL) obtained during a flexible bronchoscopy 3) Cough resolved following a 2-week course of an appropriate oral antibiotics (amoxicillin-clavulanate acid)
PBB-extended	PBB-micro or PBB-clinical requiring 4 weeks antibiotic treatment for cough resolution
Recurrent-PBB	Recurrent episodes (>3 per year) of PBB

According to the European Respiratory Society (ERS) guidelines new definition, PBB-clinical is based on all three of the following criteria: “presence of chronic (>4 weeks' duration) wet or productive cough; absence of symptoms or signs (i.e., specific cough pointers) suggestive of other causes of wet or productive cough (Table 2); cough resolution following a 2–4-week course of an appropriate oral antibiotic” (1, 4).

**Table 2:** Specific Findings associated with cough and possible diagnosis.

**Findings associated with cough**	**Possible diagnosis**
Spontaneously resolving cough, good health	Postinfectious cough
Wheezing, dry nocturnal cough, atopy, positive familiarity for asthma/allergy	Asthma
Protracted airway infections, wet cough, positive sputum/BAL culture of a respiratory pathogen	PBB, bronchiectasis
Recurrent lower airways infections, growth failure, chronic sinusitis, hemoptysis, steatorrhea	Cystic fibrosis
Persistent wet cough, digital clubbing, exertional dyspnea, chest wall deformity, auscultatory findings	CSLD^*^
Recurrent, severe or atypical Infection	Immunodeficiency (primary or secondary)
Vomiting, sialorrhea, neurodevelopmental disorders	GERD^**^–foreign body aspiration
Stridor, metallic, or biphasic cough	Airway anomalies (tracheomalacia–bronchomalacia)
Situs inversus, recurrent sinusitis, and/or otitis, recurrent lower airways infections	Primary ciliary dyskinesia

* *Chronic suppurative lung disease*.

** *Gastroesophageal reflux disease*.

The following additional definitions are used in clinical practice: PBB-extended is PBB-micro or PBB-clinical requiring 4 weeks antibiotic treatment for cough resolution; recurrent PBB is used to define recurrent episodes (>3 per year) of PBB (Table 1) (10).

According to the American College of Chest Physicians (CHEST) methodological guidelines, too, the definition of microbiologically based PBB (or PBB-micro) should be used “for children aged ≤ 14 years with PBB with lower airway (bronchoalveolar lavage or sputum) confirmation of clinically important density of respiratory bacteria (≥ 10^4^ CFU/ml),” in order to differentiate it from clinically based PBB (3).

We know that PBB is a common cause of persistent wet cough in preschool children aged 0-6 years worldwide (although sometimes it may affect even older subjects). It is diagnosed in 11-41% of children consulting a pulmonary specialist. These data are confirmed by two main studies: the first is a prospective multicenter study on the cause of chronic cough including 346 children aged <18 years (mean age of 4.5 years) recruited from five Australian major hospitals and three rural-remote clinics newly referred with chronic cough, where it was discovered that the main cause (41%) was PBB (11). The latter more recent study included 563 children aged <17 (mean age of 5.4 ± 3.8 years), admitted to the pediatric department for chronic cough. Among these patients, the most common final diagnoses were asthma (24.9%), asthma-like symptoms (19%), PBB (11.9%), and upper airway cough syndrome (9.1%) (12).

For these reason, in the clinical practice, it is important to know this lung disease in order to start an appropriate therapy before the associated complications arise.

## Clinical Features and Diagnosis

As mentioned above, the most frequent symptom in children with PBB is persistent wet cough. Generally, the median age ranges from 1.8 to 4.8 years even though PBB can occur also later (>12 years) (11). Frequently, there is neither a correlation with upper airway inflammation such as otitis or sinusitis nor signs of underlying chronic suppurative lung disease (CSLD) such as digital clubbing, chest wall deformity, and auscultatory wet sound (10, 13). The prevalence of atopic features is similar to children without PBB, and no specific correlation with the exposure to tobacco smoke has been evidenced (1, 13) Nevertheless, tobacco exposure is known to be a risk factor for the development of chronic respiratory diseases (14).

Although generally parents report wheezing, auscultatory feedback is rare and more frequently “rattling chest” and crackles are heard (10). Sometimes, the symptoms of PBB are confused with those of asthma because of similar elements, and occasionally, they could coexist. What differentiates the two pictures are mainly the type of cough (wet in PBB and often dry and/or nocturnal in asthma) and the response to antibiotic treatment in PBB.

If a child has chronic wet cough and suspected asthma not responding to rescue and background medications, empiric treatment for PBB should be assessed (8).

**Figure 1:**
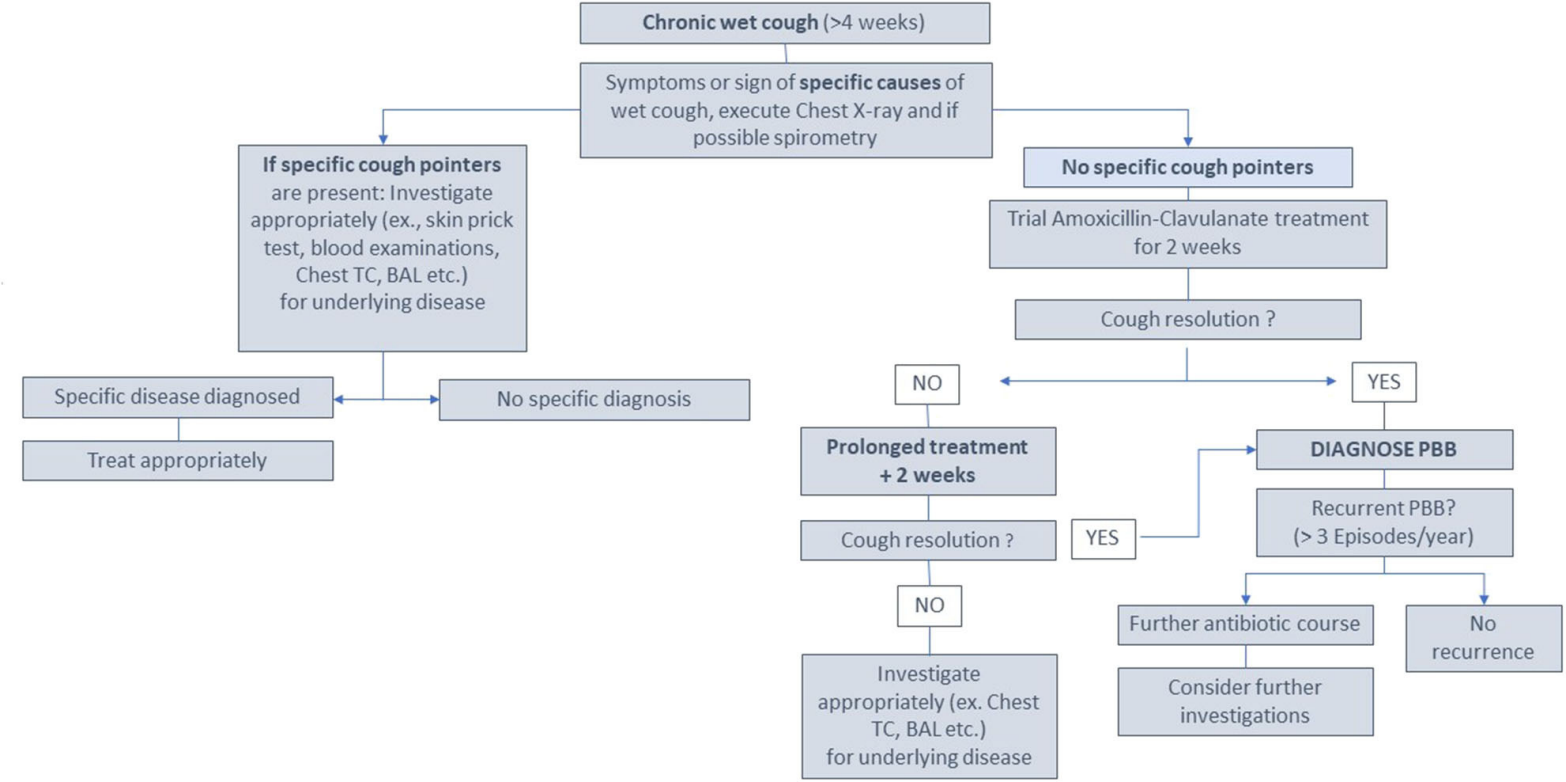
A possible approach to investigation and management of a child with chronic wet cough (>4 weeks) according to supporting evidence (1, 10, 26) (modified from Kantar A, Chang AB, Shields MD, Marchant JM, Grimwood K, Grigg J, et al. ERS statement on protracted bacterial bronchitis in children. Eur Respir J 2017;50 (2). doi: 10.1183/13993003.02139-2016).

Therefore, diagnosis of PBB is mainly clinical; generally, no instrumental examinations are needed (Figure 1). Among children undergoing chest radiograph, there are no significant changes, so the examination is normal in most cases (sometimes the chest imaging shows only peribronchial changes) (8, 15). When performed, lung functional test results are usually normal (10).

Although PBB may coexist with other diseases such as asthma, no studies assessing objective reversible airflow limitation are available to date (1).

CT scan should only be performed following a treatment failure to evaluate the possible presence of underlying bronchiectasis (8). BAL with a flexible bronchoscope from lower airways should be performed in cases of relapse after three courses of antibiotic; however, the timing is also to be assessed with the parents (16). According to ERS statement, usually BAL is carried out in the most affected lung area (identified radiologically and/or endoscopically) (17).

In infants, it is often easier to perform BAL in the right lower lobe being, along with lingula, the preferred site because these areas offer better fluid recovery (17, 18). BAL is generally not well-tolerated, and although it is a safe procedure, it may cause hypoxemia; therefore, a recent study compared BAL and bronchial aspirate (BA) to investigate if the latter would bring to similar results (19). Both BAL and BA cultures provided the same result among the majority of patients (66%). Differences affecting the choice of treatment were found just in a small number of subjects with PBB (10% overtreated, 6% undertreated, 4% would have received a different therapy). The study concludes that BAL still remains the gold standard, even though BA could be considered in cases where BAL is not tolerated, considering that the results are overlapping in the majority of cases (19).

In contrast to the above, BTS guidelines suggest that, among children with PBB, underlying conditions should be excluded, and a sputum culture should be performed before the diagnosis (5).

Likewise, when a wet cough persists after 4 weeks of appropriate antibiotics, CHEST guidelines also suggest performing “further investigations (e.g., flexible bronchoscopy with quantitative cultures and sensitivities with or without chest computed tomography)” (3).

## Etiopathogenesis

### Bacteria Pathogens and Microbiota

Several studies documented *Haemophilus influenzae* as the most common bacteria found in the BAL of subjects with PBB (47–81%), with high bacterial loads (≥10^5^ CFU/ml) (1). Most *H. influenzae* are non-typeable (NTHi) strains representing different genotypes (10). *Streptococcus pneumoniae* (24–39%) and *Moraxella catarrhalis* (19-43%) follow with variable percentages among different studies (10). Finally, it should be noted that polymicrobial infections involving more than one pathogenic bacterium have been reported in BAL of children with PBB (30-50%) (1, 20, 21).

Different studies examined the lower airway microbiota of children with PBB. The first study did not reveal significant differences regarding the microbiota composition between children with bronchiectasis, cystic fibrosis, and PBB; the core microbiota was superimposable with predominance of *H. influenzae* and oral aerobic and anaerobic (as *Prevotella melaninogenica*) (22). This contrasted with the data found among adults and suggested that a chronic airway infection starts in a similar way with inadequate airway clearance of normal microbiota, but as time passed, the microbiota in these disease groups progressively diverge from one another as a result of antibiotic drugs and (maybe) as the consequence of the underlying disease (10).

A second study conducted in 2016 found some significant differences between the microbiota of the upper and lower airways between children with PBB, bronchiectasis, and controls (23). In 2017, a team of UK researchers compared protected brushing of 20 healthy controls and 24 children with PBB, finding that the microbiota of the latter was less different in terms of richness and evenness. Bacterial communities in children with PBB were dominated by Proteobacteria, and indicator species analysis showed that *Haemophilus* and *Neisseria* were significantly associated with the patient group (24). A more recent study found out that in BAL of children with PBB, one or more respiratory pathogens were detected. Moreover, children with PBB showed that a higher BAL bacterial biomass strongly correlated with neutrophilic inflammation. PBB-microbiota were different to control-microbiota and clustered into four distinct microbiota patterns where respiratory pathogen or other microbiota species (e.g., *Prevotella*) have been detected (25). The cultures of respiratory pathogens, inflammatory markers, and BAL bacterial biomass were found to not be associated with this variation in alpha diversity among subjects with PBB. This suggest that inflammation and increased bacterial biomass in PBB cannot be caused only by single pathogenic species (25).

Differences between the data from these two recent studies may be due to different average age of the patient cohort, geographical differences, or contamination of the BAL with upper respiratory flora. Finally, a last interesting finding of the study is that *Prevotella*-associated profiles were similar to those of children with pathogen-dominated microbiota, and this could mean that even the microbiota in some cases contributes to inflammation in these patients. This could explain why some children with chronic cough and lower inflammation without respiratory species detected still respond to antibiotic treatment (26). Nevertheless, further studies are needed to establish whether a high relative load of *Prevotella* could explain the need of longer antibiotics (4 weeks) among children with PBB (10, 25). It is therefore important to understand the role of the microbiota in the pathogenesis of PBBs to identify any other bacteria involved in the recurrence and progression to bronchiectasis (25).

### Biofilm

A biofilm is “an assemblage of surface-associated microbial cells that is enclosed in an extracellular polymeric substance matrix; bacterial growth and activity are substantially enhanced by the incorporation of a surface these organisms could tie to” (27). This matrix decreases antibiotic penetration protecting bacteria against antibiotics (28).

It is reasonable to assume that a chronic bacterial bronchitis develops when one or more pathogens aggregate to form biofilms within the conducting airways dominating a niche. The prevalence of a single species or mixed populations within the biofilms drives a chronic inflammatory state, which induces a favorable environment for some bacteria such as non-typeable *H. influenzae* (NTHi) (29). Viral infection appears not only to enable the starting of both the surface attachment and of biofilms but also to be the trigger for the exacerbations characterized by the release of planktonic organism, which will generate an enhanced inflammatory response (29).

The presence of biofilms can cause the need for prolonged antibiotic therapy, and it has been detected both in BAL of children with bronchiectasis and with PBB (10).

### Virus

Different types of virus have been detected in BAL from children with PBB, but the clinical significance of this is unclear. In a first study on these subjects, high virus detection was reported in PBB patients (67%) compared to controls (38%), and the most common identified virus was Adenovirus (AdV) in the PBB-BAL (23%) compared with controls (4%) frequently encountered with *H. influenza* coinfection (13). The same study found other viruses such as Rhinovirus (41%), human Bocavirus (4%), and human Coronavirus (4%) with overlapping prevalence between the two groups (13).

There is only one study in the literature on this topic, published in 2019, whose data are opposed to those already known. This study looks for 10 common viruses in the BAL of patients with PBB and controls. The detection rate is almost the same within the two groups (23.5–28.6%) (30). Moreover, no AdV in BAL in PBB cases were detected, in contrast to previous studies (13, 30). Based on this conflicting data, nowadays, we can say that there is no certain evidence that PBB may be virus induced.

### Association With Large Airway Lesions

The correlation between major airway injuries and recurrent bronchitis is well-known (31). Airway malacia is detected frequently in children with PBB (30). It may affect airway clearance, therefore predisposing to PBB, although also airway inflammation may predispose to malacia in a vicious circle (10, 32).

Kompare et al., in their retrospective study about PBB and tracheobronchomalacia, have assessed 70 children (20 female and 50 male) with protracted cough, wheeze, and/or noisy breathing in whom BAL found ≥10^4^ CFU/ml of potentially pathogenic bacteria; children with other major conditions were excluded (asthma, cystic fibrosis, and other known chronic diseases) (33). They reported malacia in 74% of PBB cases (33).

A prospective study by Wurzel et al. on a cohort of 104 children with PBB evidenced a correlation with tracheo- and/or bronchomalacia in 68% of (13).

Furthermore, airway malacia can both decrease effectiveness of cough and interfere with normal mucous movement, a crucial mechanism for clearing bacteria from the airways (34).

As postulated by Donnelly et al., airway malacia could induce an impairment of normal pulmonary defense mechanisms promoting the development of chronic cough and PBB (21).

### Immunity and Inflammation

Children with PBB usually do not have immunodeficiencies; therefore, most of them have normal serum immunoglobulin levels by age (IgG, IgA, IgM, and IgE) as well as normal antibody-mediated response to protein (tetanus) and conjugated protein-polysaccharide (*H. influenzae* type b) (9, 13).

Nevertheless, several studies report the presence of intense airway neutrophilia with a neutrophil percentage between 25.5 and 44%; no eosinophilia was found, and just one study reported percentage increase in lymphocytes (9, 21, 35, 36). Lymphocyte subsets were normal, except for increased CD56 and CD16 natural killer cell level for age, probably associated with recent viral infection (1, 13, 37). Increased levels of interleukin (IL)-8, IL-1β, and active matrix metalloproteinase-9 seems to correlate with the degree of neutrophilia (38).

A study of Chang et al. detected increased human β-defensin-2 and mannose-binding lectin levels, while activated caspase-1-dependent proinflammatory pathways in response to NTHi were also identified in pediatric patients with PBB, because both the innate pathogen recognition and clearance mechanisms were normal (36). In addition, higher levels of Toll-like-receptor 2 (TLR-2) and Toll-like-receptor 4 (TLR-4) in the BAL of children with PBB are reported compared to controls (38).

Finally, another study focuses on the possibility of an impaired clearance of apoptotic cells by alveolar macrophages (efferocytosis). The remaining apoptotic cells may undergo secondary necrosis with proinflammatory effect, thus increasing chronic inflammation and tissue damage (10, 39).

### Overlap With Bronchiectasis

In 2008, Chang et al. proposed a paradigm where “PBB, CSLD and bronchiectasis shared common underlying pathobiological mechanisms and progressed variably along an increasing spectrum of severity” (8). The similarities are chronic wet cough, rattling breathing, defective mucociliary clearance, endobronchial bacterial infection, and neutrophilic airway inflammation (10). The major differences between these conditions consist in the clinical severity, the improvement to 2–4 weeks of adequate antibiotic treatment, chest high-resolution CT scan findings, and subsequent management (10, 40).

Starting from these observations, analysis were made to address the possible existence of more elements predicting the evolution of PBB in bronchiectasis capable of explaining why among children with recurrent PBB, some subjects do not show pulmonary sequelae, while other children develop bronchiectasis. A recent prospective longitudinal cohort study assessed the 2-year outcomes of 161 pediatric patients with PBB and detected two main risk factors for bronchiectasis: recurrent PBB (>3 episodes/years) and a positive BAL culture for *H. influenzae*. This finding correlated with a higher risk of bronchiectasis (more than seven times) compared with no infection (41). Moreover, authors showed that ~1 of 12 children with PBB are diagnosed with bronchiectasis at 2 years follow-up, with many experiencing recurrent episodes of PBB. This study provides further evidence to support a link between PBB and bronchiectasis in young children. This may also suggest the need to monitor children with PBB over time and to consider chest CT imaging in those with risk factors for bronchiectasis (41).

Noteworthy are some evidence showing that lower airways of PBB and bronchiectasis are characterized by marked neutrophilic inflammation with intense proinflammatory mediator responses such as interleukin-8, matrix metalloproteinase-9, and IL-1β. All these findings significantly differ to controls and support the hypothesis that lower airway microbiology and pathobiological aspects are similar in PBB and bronchiectasis (35, 38).

According to the 2017 CHEST guideline and expert panel report, there is high-quality evidence that the administration of appropriate antibiotics among children aged ≤ 14 years with wet/productive cough improves cough resolution, although further investigation should be undertaken when specific cough pointers (e.g., digital clubbing) are present. When the wet cough does not improve in response to 4 weeks of antibiotic therapy, there is moderate-quality evidence that further investigations such as flexible bronchoscopy, chest CT scan, and immunity tests should be considered to look for an underlying disease (3).

## Management

Children with PBB should be treated with antibiotic for at least 2 weeks. Several studies have been performed, and use of prolonged antibiotic treatment has been shown to facilitate cough resolution compared to placebo (42).

Specifically in a randomized controlled trial conducted by Marchant et al. (26), including 50 children (median age, 1.9 years) with chronic wet cough (>3 weeks) a 2-week treatment with amoxicillin-clavulanate acid allowed cough resolution compared with placebo (48 vs. 16%) (26). Amoxicillin-clavulanate acid is the most commonly used antibiotic due to its activity against β-lactamase, although other options such as oral second or third generation cephalosporins, trimethoprim-sulfamethoxazole, or a macrolide may be used among patients with IgE-mediated reaction to penicillin (1). Nevertheless, oral cephalosporins because of its similarity to penicillins (e.g., ampicillin and cefalexin or cefaclor) should be avoided among these patients (1). Some children require up to 4 weeks of treatment. Marchant J. et al. in their randomized controlled trial (RCT) cited above shows that many of the children not responding after 2 weeks of treatment had underlying tracheobronchomalacia (26), even though better evidence is needed to determine whether a prolonged course of antibiotics is beneficial, due to the inherent risk of antibiotic therapy. Furthermore, during a different study on 144 children eligible upon defined criteria [presence of chronic wet cough >4 weeks and having completed at least 4 weeks of oral antibiotics directed against likely respiratory bacterial pathogens associated with PBB, cystic lung diseases (CLDs), and bronchiectasis] Goyal V. et al. showed that children affected by chronic wet cough not improving after 4 weeks of appropriate treatment have increased likelihood (88/105, 83.8%) of bronchiectasis on a chest CT scan (43). Some clinicians prefer to use prolonged therapies even beyond the resolution of symptoms, and the rationale is that protecting the airways against the common respiratory bacteria for a longer period reduces the risk of reoccurrence and recovers airways integrity (1). However, prolonged antibiotic treatments may cause dysbiosis and the selection of antibiotic-resistant strains (44). Lastly, the role of 1-weekly azithromycin in PBB is not clear even though it seems halving the rate of exacerbations in children with either CSLD or bronchiectasis (1, 45).

BTS cough guidelines suggest that all children with PBB should receive both 4–6 weeks of antibiotics (5) and physiotherapy.

## Conclusions

PBB is a common cause of persistent wet cough in preschool children worldwide; it is frequently underdiagnosed or mistaken for other diseases such as postviral cough or asthma and therefore inadequately treated.

Considering PBB in the differential diagnosis of chronic wet cough in children allows an early and adequate antibiotic treatment to eradicate the infection. BAL and antibiogram generally are not required, as well as chest X-ray, and clinical diagnosis is enough to start an empiric 2 weeks therapy with amoxicillin-clavulanate acid. This therapy is mostly effective against the bacterial species involved, such as *H. influenzae* (NTHi), *S. pneumoniae*, and *M. catarrhalis*. Moreover, an adequate therapy prevents the onset of a prolonged inflammatory process potentially associated with structural damage of the lower airways, which may be involved in bronchiectasis. Several studies provide evidences to support a link between PBB and bronchiectasis in children, and this emphasizes the necessity to consider this possibility of evolution and to carry out some more detailed investigations in the case of a clinical suspicion.

## Author Contributions

MP and MG performed the literature review. GR coordinated the writing group. All authors critically reviewed the manuscript and read and approved the final version.

## Conflict of Interest

The authors declare that the research was conducted in the absence of any commercial or financial relationships that could be construed as a potential conflict of interest.

## Abbreviations

BA: bronchial aspirate
BAL: bronchoalveolar lavage
CLDS: cystic lung diseases
CT: computerized tomography
GER: gastroesophageal reflux
NTHi: *Haemophilus influenzae* non-typeable
PBB: protracted bacterial bronchitis
QoL: quality of life
UACS: upper airway cough syndrome.
